# Inflammation and Progression of Cholangiocarcinoma: Role of Angiogenic and Lymphangiogenic Mechanisms

**DOI:** 10.3389/fmed.2019.00293

**Published:** 2019-12-18

**Authors:** Sukanya Roy, Shannon Glaser, Sanjukta Chakraborty

**Affiliations:** Department of Medical Physiology, Texas A&M Health Science Center College of Medicine, Bryan, TX, United States

**Keywords:** microenvironment, inflammatory signaling, cholangiocarcinoma, tumor lymphangiogenesis, angiogenesis

## Abstract

Cholangiocarcinoma (CCA), or cancer of the biliary epithelium is a relatively rare but aggressive form of biliary duct cancer which has a 5-year survival rate post metastasis of 2%. Although a number of risk factors are established for CCA growth and progression, a careful evaluation of the existing literature on CCA reveals that an inflammatory environment near the biliary tree is the most common causal link between the risk factors and the development of CCA. The fact that inflammation predisposes affected individuals to CCA is further bolstered by multiple observations where the presence and maintenance of an inflammatory microenvironment at the site of the primary tumor plays a significant role in the development and metastasis of CCA. In addition, mechanisms activating the tumor vasculature and enhancing angiogenesis and lymphangiogenesis significantly contribute to CCA aggressiveness and metastasis. This review aims to address the role of an inflammatory microenvironment-CCA crosstalk and will present the basic concepts, observations, and current perspectives from recent research studies in the field of tumor stroma of CCA.

## Introduction

Cholangiocarcinoma (CCA) is a term used to define a group of different biliary epithelial cancers and is the second most common type of liver cancer. This group of primary biliary malignancy represents three different classically recognized kinds of biliary tree cancers, classified on the basis of anatomical point of origin in the bile duct, intrahepatic CCA (iCCA), perihilar CCA (pCCA), and distal CCA (dCCA) (1). Among these three types, iCCA originates in the intrahepatic ducts and represents the second most prevalent type of primary liver malignancy (about 10% of primary liver malignancies are iCCA). Duration of survival post-resection in intrahepatic CCA is 12.4 months (2). The most common type of CCA, is the pCCA, constituting ~50–60% of all recorded cases. pCCA comprises tumor arising from the emergence of left/right hepatic ducts at liver hilum to the confluence of cystic duct with common hepatic duct (choledocus formation) while distal CCA representing 20–30% of CCA occurs in the epithelial cells of the extra hepatic bile ducts (3). Although iCCA represents only about 5–10% of all CCA cases there is an increase in the number of iCCA among the three CCA types being observed recently (4). Internationally, CCA cases have increased since the past decade, in United States ~5,000 new cases are diagnosed each year (5). The incidence of CCA is highest among Hispanics and Asians (2.8–3.3/100,000) and lowest (2.1/100,000) among non-hispanics and African Americans (6). With a 5 year mortality rate post metastasis of 2%, CCA, originally described as a rare form of cancer is receiving more attention compared to the past decades due to its high mortality rate (1).

## Etiology

The number of people afflicted with CCA differs geographically primarily because of the difference in the presence of the risk factors that predispose an individual toward CCA. The number of CCA cases is higher in Asian countries (7). CCA also shows a slight bias toward the male gender (7). The number of risk factors and their extent of influence on CCA predisposition, is not as high as in other malignancies. This could be partially due to the limited number of studies focused on identifying risk factors of CCA. Presence of bile duct cysts, primary sclerosing cholangitis, liver cirrhosis, hepatobiliary parasitic infections such as with liver fluke, hepatolithiasis, and thorotrast exposure, are the most common risk factors. Further complicating this scenario is the fact that a majority of CCA cases develop without the presence of any of the above-mentioned risk factors (8). Hence, there is a need to look at new prognostic factors that will aid in predicting the surgical eligibility, outcome and survival of CCA patients. This has opened up new avenues in research and has identified the critical role of different inflammatory cytokines, increased lymphangiogenesis, relatively low angiogenesis, cancer associated fibroblasts (CAFs), mesenchymal stem cells (MSCs), and other factors, in the growth and progression of the different types of CCA. In the next section we will review some of these risk factors.

### Primary Sclerosing Cholangitis (PSC)

Inflammation and inflammatory mediators form a key underlying basis for several risk factors significantly associated with CCA (9). An inflammatory and obstructive autoimmune disease of the bile ducts, PSC is one of the most important risk factors of CCA. Patients with PSC have a 400-fold higher chance of developing CCA than those without PSC. Interestingly, majority of PSC patients are between the ages of 30 and 40 (in general CCA has a reported age specificity of 60–70 years in age) at the time of diagnosis (9). Up to 50% of these cases are recorded in the first year of diagnosis of PSC (10). The presence of chronic inflammation of bile ducts typically associated with PSC is thought to be one of the reasons for this heightened risk (11–13). Other factors that serve as the link between PSC and CCA include increased proliferation of the epithelial cells of the biliary tree, cholestasis in the ducts leading to the liver and presence of mutagens produced in the bile (10, 11). The role of inflammation in the growth and rapid development of CCA is also underscored by studies that identify inflammatory bowel disease (IBD) as one of the risk factors of CCA (12, 13).

### Cirrhosis

Liver cirrhosis develops as a consequence of liver diseases and/or conditions such as alcoholism and hepatitis. As a result, the liver parenchyma is dominated by fibrosis/scarring of liver tissue resulting in disruption and eventual loss of normal liver function. Cirrhosis is an important risk factor for iCCA (14) and shows high degree of association, especially, in Asian populations (15). Similar to PSC, this too has an inflammatory stimulus and a sudden rise in epithelial proliferation, presence of pro-inflammatory cytokines and chemokines and the generation of fibrotic nodules in liver, mediates a link between cirrhosis and CCA (3).

### Liver Fluke Infections

Liver fluke (*Clonorchis sinensis, Opisthorchis viverrini*) infections have been identified as critical risk factors for CCA especially in eastern Asian countries where these infections are deemed to be endemic (16). In fact, the recognition of *O. viverrini* as a cancer-inducing parasite by IARC (International Agency for research on Cancer) is due to its role in the development of CCA in affected individuals. These infections are associated with a rise in inflammation, generation of fibrotic nodules, obstruction of bile ducts and/or cholestasis. Chronic inflammation in the biliary tree in *O. viverrini* infected patients (especially in the background of gene polymorphisms and exposure to other environmental factors) leads to CCA development (17, 18).

### Viral Infections

Viral infections such as hepatitis B and C (HBV and HCV, respectively), serve as important risk factors for CCA (19). While HBV infection is endemic to Asian countries and thus serves as the stronger risk factor for iCCA (20), HCV is the primary causative agent for iCCA in western countries (14). Cirrhosis is a common manifestation of hepatitis and leads to the development of the chronic inflammatory background that predisposes to CCA. However, the role of hepatitis viruses in causing proliferation of the hepatic epithelium is also considered to be a reason for CCA incidence in hepatitis patients (21).

### Choledocholithiasis and Hepatolithiasis

Choledolithiasis and cholelithiasis are conditions that involve the presence of stones in the gall bladder and common bile ducts. The presence of these gall stones causes biliary obstruction resulting in cholestasis and serve especially as a risk factor for extrahepatic CCA (22). The presence of stones or calcium deposition inside the intrahepatic bile ducts also leads to cholestasis and chronic inflammation, ultimately serving as a risk factor for CCA. In the Asian population, 5–13% of patients with hepatolithiasis develop iCCA (15, 23).

### Other Inflammatory Conditions

Chronic pancreatitis is a strong risk factor for extrahepatic CCA with an odds ratio of 6.61 (95% CI 5.21–8.40) in comparison to the 2.66 odds ratio of iCCA (95% CI, 1.72–4.10). In chronic pancreatitis too, cholestasis and inflammation may arise leading to CCA (24). Also, the presence of cysts in the bile ducts (intrahepatic and extrahepatic) when left untreated leads to the development of iCCA and eCCA tumors, because of biliary duct obstruction and dilatation leading to cholestasis and inflammation (24, 25). Thus, it is evident that inflammation forms an underlying theme in the predisposition and development of CCA.

## Cellular Origin of CCA

### Role of Cell of Origin

As CCAs originate from cholangiocytes from different anatomical locations of the biliary tree, they also exhibit considerable tumor heterogeneity that points to the possibility of diverse cellular origins (1, 26). In general, CCA originates from the peribiliary gland (PBG) lining epithelium of intra- and extra- hepatic ducts (IH and EH, respectively) of the biliary tree (27). Additionally, cholangiocytes and hepatocytes originating from canals of Herring can undergo mutation to give rise to tumors having varying phenotypes (28). Based on the wide range of these phenotypes, pCCA and dCCA have been characterized as adenocarcinomas mucinous in nature, while iCCA has two subtypes: iCCA arising from small bile ducts, mixed in histological phenotype and those arising directly from large intrahepatic bile duct, mucinous in histology (29, 30). While bile ductular type iCCA has been recognized to be associated with solid tumor formation not having preneoplastic lesions, iCCA arising from large intrahepatic bile duct is one which is distinctly preceded by preneoplastic lesions (biliary intraepithelial/intraductal papillary neoplasm). Additionally, bile ductular type iCCA has been correlated with chronic liver disease cases such as cirrhosis in contrast to bile duct type iCCA which is mostly correlated with PSC. These differences in histology point to the role of different cells of origin of CCA (29, 30).

### Stem Cell/Progenitor Niches for CCA Development

The undeniable role of stem cells in CCA development and origin is proven by the fact that human hepatic stem cells (hHPSCs) are the progenitor cells giving rise to cholangiocytes and hepatocytes that mutate to give rise to CCA (28). The PBG niche starts at septal-segmental bile ducts and ends near duodenal area at hepatic pancreatic common duct. PBG niche thus distributed all across the biliary tree has a significant role in harboring a multipotent stem cell niche which forms the source of the endodermal hepatic mucinous cells that ultimately give rise to the mucinous CCA subtypes of dCCA, pCCA and bile duct type iCCA (27, 30–32). Cancer stem cells (CSCs) are more generally characterized as cellular subset that maintains tumor growth, such CSCs are recognized by the expression of extracellular markers like CD 24, CD44, CD133, epithelial cell adhesion molecule (EpCAM) etc. in liver malignancies (33). In CCA, more of these studies identifying the specific roles of CSCs are needed. As such two distinct stem cell niches are recognized for CCA development: BTSCs (biliary tree stem cell niche within PBG) and hHPSCs within canals of Herring (26, 27). These findings suggest that CCA has more than one type of cell-of-origin and the differences can be looked at to develop a treatment strategy(s) that is personified from an anatomical point of view (34).

## Factors influencing the inflammatory tumor microenvironment of CCA

CCA is one of the most desmoplastic tumors and the tumor microenvironment of CCA is characterized by a dense bed of connective tissue intertwining the tumor cells. This dense stroma is composed of a contiguously activated subset of fibroblasts called CAFs that play key roles in modulating several aspects of CCA progression (35). Further during tumor development and progression and resulting increase in cellular and metabolic demands there is often restricted access to nutrients and oxygen supply. This results in regions of the solid tumor having permanent or transient hypoxia, due to alterations in the tumor associated vasculature (36). The expanding vascular network is unable to meet up with the growing demands of the tumor and hypoxic regions persist and induce cellular pathways that promote more malignant phenotypes. In addition, there are immune cells, blood vessels, and lymphatic vessels that contribute to tumor progression which will be discussed in the following sections.

### Role of Cancer Associated Fibroblasts

CAFs release a number of molecules functioning as extracellular matrix proteins (ECM) such as collagen I and fibronectin (35). In CCA, CAFs typically infiltrate the tumor stroma, and are differentially stimulated by a variety of molecular factors released by CCA tumor cells as well as hypoxia. The CAFs population in CCA thus is heterogenous in origin (37). Two of the main sources of these CAFs are liver (hepatic stellate cells, HSCs) and portal vein (portal fibroblasts), while bone marrow derived MSCs also serve as a source of CAFs to a minor extent (37). CCA tumor cells and other immune cells such as macrophages secrete inflammatory chemokines, cytokines and growth factors that not only signal fibroblasts from liver and portal vein to infiltrate the tumor microenvironment but also result in constitutive activation of fibroblasts (35). Platelet derived growth factor (PDGF-DD) overexpressed by CCA cells under hypoxic condition has been shown to be an important CAF infiltrating factor. Binding of PDGF-DD to its receptor PDGFRβ activates Cdc42, Rac1, and Rho GTPases and JNK pathways (38). PDGF-DD binding Cdc42 induces the formation of filopodia and Rac1 induces the formation of lamellipoda, thus ensuring the migration of CAFs to CCA tumor stroma. In addition to PDGF-DD, a number of other growth factors such as FGF (fibroblast growth factor), numerous factors belonging to PDGF family and TGF-β also aid CAF infiltration (39).

Alpha-smooth muscle actin-positive (α-SMA) fibroblasts promote biliary cell proliferation and correlate with poor survival in CCA. CCA fibroblasts have proliferative effects that enhance tumor promotion and progression of CCA (40). CCA patients with a high population of CAFs have poorer prognosis than patients with low number of CAFs (41). Consequently, CAF-specific α-SMA is a prognostic factor of CCA patient survival (42). The tumor boosting ability of stromal CAFs was also shown using a 3D collagen matrix-based co-culturing system, in which CCA cells and CAFs isolated from a syngeneic orthotopic rat model of CCA showed a corresponding increase in the formation of structures resembling ducts from CCA cells with the increase in CAF plating density (43). Interestingly, hepatic stellate cells (HSCs) under the influence of CCA cells can also transform into CAFs and support CCA growth (44, 45). These findings were further corroborated by studies in a syngeneic rat CCA model with selective stromal CAF depletion that exhibited improved host survival and decreased tumor growth (46).

#### Factors Supporting CAF-CCA Cross-Talk

CAFs in CCA show unique characteristics and gene signatures (47). Gene expression studies with human CCA sample derived CAFs showed significant differences between normal liver fibroblasts and CAFs. Most of the genes that were induced in CAFs were involved in controlling cellular metabolism, a prerequisite for the active production of cellular proteins to support the tumor microenvironment and promote tumorigenesis (47). In addition, exosomes also serve as important vessels for transporting regulatory molecular factors (between CAFs and CCA cells) thus supporting cross-talk between CCA cells and CAFs. While studies characterizing the exosomal cargo involved in CAF-CCA crosstalk has been relatively limited (48, 49), it has been shown that exosomes shuttle miR-195 between CAFs and CCA (50). Stimulation of MSCs to CCA cell-derived exosomes lead to increased migration and production of inflammatory tumor promoting cytokines as CXCL1, CCL2, and IL-6 (51). In addition, several growth factors contribute to the inflammatory microenvironment.

EGFA/EGFR binding has been shown to promote tumorigenesis and metastasis in CCA, another important EGFR ligand, HB-EGF was found to be highly expressed in myofibroblasts. HB-EGF activated EGF signaling promotes proliferation of CCA cells and also induces epithelial-mesenchymal (EMT) changes as well as invasion. HB-EGF secretion from fibroblasts is also activated by the pro-tumorigenic growth factor TGF-β secreted by tumor cells that in turn favors CCA growth (52).

Stromal cell derived factor 1 or SDF-1 has previously been reported to be involved in promoting cancer growth as a ligand for CXCR4/CXCR7 (53). In CCA, SDF-1 expression is only produced by the stromal CAF, possibly as a result of the HSC infiltration under stimulatory signals derived from angiotensin-II secreted by cancer cells (54). *In vitro* studies indicate that when SDF-1 is expressed by HSCs, a number of pro-tumorigenic responses are induced such Bcl-2, and activation of PI3K/Akt pathway. These responses initiate increased CCA cell invasion and prolonged survival in addition to inducing epithelial-mesenchymal transition (45, 55). Tumor associated macrophages were shown to produce TNF-α that induces CXCR4 expression, thus promoting SDF-1 mediated pro-tumorigenic effects (54). CAFs are also shown to release high levels of HGF (hepatocyte growth factor) that might mediate high expression of CXCR4 (43).

### Role of Mesenchymal Stem Cells (MSCs)

One of the most important cellular components of CCA stroma are MSCs. MSCs may activate a series of tumor signaling pathways through the release of cytokines and that may either promote or inhibit tumor development and progression (56). The function of MSCs in tissue repair is similar to the homing of MSCs to sites of tissue damage and to sites of tumor microenvironment (51). Injured tissues secrete a wide variety of inflammatory chemokines that sends signals to MSCs for repair. It has been seen in a number of studies that tumor cells too, while modulating several other factors in their microenvironment that foster a metastatic condition, secrete inflammatory chemokines that result in MSC infiltration (51). CCA cells also secrete exosomal vesicles that are shown to enhance expression of IL-6, CXCL-1, and CCL2 by MSCs. Further, conditioned medium from MSCs exposed to tumor cell-derived extracellular vesicles (EVs) caused an upregulation in STAT3 phosphorylation and proliferation of CCA cells, possibly by secretion of CCL2/MCP1, CXCL1/GRO-α, CXC3CL1/Fractalkine, IL-6, and PDGF-AA (51). Conditioned media from MSCs also has been found to upregulate the Wnt signaling pathway in CCA cells and increased nuclear translocation of ß-catenin (57). Further, coculture studies of CCA and MSCs have shown that increased CCR5 expression by tumor cells upregulates metalloproteinases MMP-2 and MMP-9 in CCA cells and thereby promoted angiogenesis and CCA metastasis (58).

### Role of Macrophages

The CCA stroma is densely populated by different infiltrating immune cells among which tumor associated macrophages (TAMs) play an important role by regulating angiogenesis, lymphangiogenesis, tumor proliferation and also modulating matrix related changes (59, 60). In a study by Wongkham et al. more than half of CCA tumor samples showed high macrophage infiltration in CCA (61). It has also been seen that CD14^+^/CD16^+^ monocyte cells which are precursors of tissue resident macrophages are present in an increased number in CCA patients. It is significant that these circulating CD14^+^/CD16^+^ monocytes have high VEGF and CXCL3 expression that promote tumor angiogenesis (62). In a correlation study it was seen that CD163^+^ M2 macrophages were associated with FOXP3^+^ regulatory T cell-related infiltration. Additionally, this study also showed that CCA conditioned media treatment of macrophages led to polarization bias toward M2 macrophages along with secretion of TGFβ, IL10, and VEGF-A (63). A high density of the M2-TAMs in patients is significantly associated with increased extrahepatic metastases possibly due to the effects on EMT pathways (41).

## Role of inflammatory cytokines

The association between chronic inflammation and the development and progression of malignancy is significantly pronounced in onset and development of CCA (64). Inflammation in the tumor microenvironment of CCA is promoted by a number of cytokines and chemokines that further enhance tumor progression and aid pathways involved in distant metastasis (47). Below, we discuss several inflammatory cytokines that contribute to an inflammatory tumor microenvironment and enhance CCA progression.

### Tumor Necrosis Factor-Alpha (TNF-α)

TNF-α is one of the most well-known mediators of inflammatory stimuli in the tumor microenvironment (65). Although TNF-α is involved in cancer progression, its more prominent pro-tumoral effects have been seen in angiogenesis and invasion of cancer cells (66, 67). During pathogenesis, TNF-α elicits an immune response at tissue injury locations. TNF-α also induces hepatic stellate cells (HSCs) so that they secrete oxidative radicals such as hydroxyl radical, nitic oxide (NO), and superoxide anion and is associated with aggressive development of CCA (68). Suksawat et al. showed that CCA cells express very high levels of eNOS and phosphorylated eNOS that correlate with poor prognosis in CCA patients. This phosphorylation mediated activation of eNOS by VEGF-C is through activation of PI3K/AKT pathway. The downstream effects of eNOS/peNOS/iNOS is thought to originate from VEGF-C pathway activation (69). TNF-α has been shown to promote migration of CCA cells by upregulating expression of S100A4, vimentin and ZEB2, molecules involved in EMT transition. In neoplastic bile ducts, these molecules have been seen to be associated with upregulation of TGF-β and downregulation of E-cadherin expression, an observation that has been correlated to poor prognosis in CCA patients (70).

### Interleukin 1β (IL-1β)

Classified as one of the most important pro-inflammatory cytokines, IL-1β has been shown to be highly expressed from HSCs. The autocrine signaling mediated by CCA cells also becomes prominent in this regard as CCA cells have been shown to produce high levels of IL-1β that further enhances the CXCL5/CXCR2 pathway that in turn activates AKT/PI3K or ERK1/2 pathways. In fact, heightened CXCL5 expression has been seen to indicate poor rates of survival in CCA patients (71, 72).

### Interleukin 6 (IL-6)

Bone marrow derived MSCs (BM-MSC) when exposed to tumor conditioned medium can transform into CAFs and stimulate tumor growth via secretion of inflammatory cytokine IL-6 in the tumor stroma. In CCA, this IL-6 overexpression was found to decrease the methylation of the EGFR promoter and enhance EGFR expression that in turn is associated with poor prognosis and overall survival (64, 73). IL-6 also mediates its tumorigenic effects by causing hypermethylation based silencing of tumor suppressor genes (74). In CCA, IL-6 has been shown to activate the p38 pathway and consequently downregulate p21^WAF/CIP1^ a cyclin dependent kinase inhibitor, involved in cell cycle regulation (75). IL-6 also induces upregulation of STAT3 and Mcl-1 (myeloid cell leukemia-1) genes that mediate an anti-apoptotic response in neoplastic cholangiocytes (76). In addition, IL-6 also induces EMT by increasing expression of Snail and JAK/STAT and a resulting downregulation of E-cadherin and promotes CCA progression (77).

### Transforming Growth Factor (TGF-β)

TGF-β plays dual roles in cancer progression and inhibits cell proliferation, regulates anti-inflammatory, and pro-apoptotic effects in cells under normal physiological conditions (78). It also actively promotes tumor progression and most cancer cells are resistant to its anti-proliferative effects. TGF-β activates the expression of its downstream genes (such as Bim) through differential phosphorylation and nuclear translocation of SMAD transcription factors (79). Mutational changes in the TGF-β receptor resulting in changes in Smad4 phosphorylation, increased cyclin D1 levels activate pathways that make CCA cells resistant to the tumor suppressive effects of TGF-β (80). Mouse model-based studies have shown that loss of expression of PTEN and SMAD4 gives rise to CCA (81). Correlation studies have shown that high levels of TGF- β is related to CCA metastasis to lymph nodes and distant sites as well as CCA recurrence (82). Consequently, inhibition of TGF-β resulted in significant reduction of CCA cell invasion (83). Further, altered TGF-β signaling in CCA cells also causes EMT-driven changes in cytoskeletal structure and CCA cell motility thus influencing cancer cell invasion through upregulation of EMT genes (84).

Overall, inflammatory cytokines set the stage for CCA growth by enhancing proliferation, activation of tumor promoting mechanisms such as EMT, activation of signaling pathways that promote tumor growth and loss of cell cycle checkpoints. However, the major cause for the high mortality associated with these cancers is its ability to metastasize, that is aided by the activation of lymphangiogenic (growth of new lymphatic vessels) and angiogenic (growth of new blood vessels). The various growth factors secreted by CCA cells into their stroma and other components of the tumor microenvironment foster the development of new lymphatic and blood vessels that in turn promote tumor growth and dissemination to distant organs.

## Lymphangiogenesis and Angiogenic mechanisms in CCA progression and metastasis

Tumor cells employ several mechanisms to establish a functional and integrated vascular system comprised of both blood and lymphatic vessels to promote cellular growth and metabolism. Expansion of these vascular networks is key to migration of the tumor cells to distant sites where they establish tumor niches. A surge of recent data has implicated the roles of both lymphatic and the blood vascular in promoting CCA metastasis.

### Lymphangiogenesis and Lymph Node Remodeling

Tumor-associated lymphangiogenesis, or the sprouting of new lymphatic vessels in the tumor microenvironment is a form of tumor-associated neovascularization that has been the focus of studies concerning the metastatic spread of highly aggressive form of cancers (85). Lymphatic involvement has emerged as a hallmark of CAA with significant lymphatic invasion or lymph node metastasis implicated with poor disease prognosis (86, 87). Early metastatic CCA is characterized by a striking expansion of the intratumoral and peritumoral lymphatic vessels, which represents a key determinant of the early metastasis to the regional lymph nodes in patients rendering patients unable to opt for surgical resection. Post-surgical resection period is characterized by an increase in lymphangiogenesis and lymphatic vessel remodeling that correlates with poor post-surgical survival (86). Hence, it is critical to look at the elements in the tumor microenvironment of CCA that cause lymphangiogenesis and lymph node remodeling.

As discussed above, the tumor microenvironment of CCA is enriched with abundant cytokines and chemokines necessary for paracrine signaling that promotes development of a lymphatic bed dedicated to sustaining the growth of tumor. CAFs actively crosstalk with CCA cells in driving the development of a rich lymphatic vasculature within a pro-lymphangiogenic tumor stroma (35). High expression of VEGF-C and VEGFR-3 has been observed in the tumor microenvironment of intrahepatic CCA patients (iCCA), that also correlated with poor prognosis in patients (88–90). VEGF-C is required for the growth of small (or intial) lymphatic vessels whereas angiopoietin 1 & 2 are need by VEGF-C to form terminal lymphatic vessels in the adult body (91, 92). The dense network of lymphatic vessels and a reduced number of blood vessels, in the CCA tumor stroma also creates a hypoxic microenvironment (93). Hypoxia inducible factor-1 (HIF-1α) is known to induce lymphangiogenesis in several cancers (94). In CCA, high expression of HIF-1α promotes tumor progression and metastasis and is associated with poor patient survival (95). Interestingly, HIF-1α has also been shown to support cancer related lymphangiogenesis by upregulating the expression and subsequent secretion of Ang1/2, VEGF-C/D and PDGF-B from neoplastic cells into the tumor stroma, in several cancers as breast cancer, esophageal cancer, and oral squamous carcinoma (96–98). PDGF-D secreted from neoplastic CCA cells binds PDGFRβ on CAFs resulting in activation of ERK/NF-kB and JNK signaling networks that in turn secretes VEGF-C and promotes expansion of the lymphatic vasculature and tumor cell intravasation. Pharmacological depletion of CAFs in a CCA *in vivo* however, significantly reduced lymphatic vascularization and reduced lymph node metastases (99). VEGF-C expression in CCA is also mediated by M2 macrophages (63). Further, overexpression of Nerve Growth Factor Beta (NGF-B) overexpression correlated with VEGF-C overexpression, lymphatic vessel density and lymph node metastasis along with nerve cell invasion in patients of hilar CCA (100). Different correlation studies have established lymphatic vessel density (LVD) and expression of several lymphatic specific markers such as podoplanin and VEGFR-3 as prognostic biomarkers of CCA (101, 102). Podoplanin is highly expressed on the surface of CAFs as well as LECs and emerged to be a prognostic biomarker in human perihilar CCA (101). Lymph node metastasis has also been correlated with a high podoplanin expression on activated CAFs in intrahepatic CCA (90). Further studies are needed to determine the role of podoplanin in tumor lymphangiogenesis in CCA. However, podoplanin mediated regulation of small GTPases as Cdc42 induces capillary morphogenesis, polarized migration, and invasiveness of LECs (103, 104).

Thelen et al. have demonstrated that a high lymphatic vessel density or existence of lymphangiogenesis significantly correlates with poor prognosis in patients with hilar CCA. This observation adds to the role of lymphatic vessel remodeling in cancer progression, specifically the migration of cancer cells via lymphatic vessels (104, 105). In CCA, a “high” LVD is associated with increased nodal spread, and “high” LVD tumors more frequently develop recurrence (105). Indeed recent studies have shown that both peritumoral as well as intratumoral lymphatic bed is composed of capillaries that lack organization and/or drainage function thus favoring neoplastic cell infiltration because of the differential permeability or leaky nature of these vessels (106). In this regard, LECs lining these vessels also interact with tumor cells to transport them through endothelium, an event mediated by the CCL1-CCR8 chemokine axis. CC-type chemokine ligand 1 (CCL1) is expressed on the surface of LECs which bind CC-type chemokine receptor 8 (CCR8) on the surface of tumor cells and thus help in their trans-endothelial migration (107). Tumor lymphangiogenesis, which results in proliferation of LECs also functions in immune-evasion of the cancer cells. LECs in draining lymph nodes express on their surface the well-known antigen PD-L1 which binds to PD-1 on the surface of cancer specific CD 8^+^ cells and induces their apoptosis (108). However, there is a need to study the mechanisms of lymph node remodeling and lymphatic metastasis in CCA, that would further establish the link between lymphatic vessel remodeling, tumor stroma, tumor lymphangiogenesis and CCA metastasis.

### Angiogenesis

Tumor related angiogenesis, or the sprouting of new blood vessels is one of the key mechanisms for tumor metastasis that is promoted by angiogenic factors actively secreted by tumor cells (109). Tumor angiogenesis is pronounced in CCA, one of the most aggressive and metastatic cancers. Cholangiocytes promote neo-vascularization by enhanced expression of pro-angiogenic growth factors both at the site of primary tumor as well as in the tumor stroma of distant sites where these cholangiocytes have metastasized. Thus, a sprawling network of blood vessels created by secreted factors from cholangiocytes supports the growth and spread of cholangiocytes (110). Critical mediators and activators of angiogenesis include the growth hormones VEGF, EGF, and NGF, FGF, placental growth factor, the angiopoietins and their receptors, Tie1 and Tie2. Further, neuropilin, ephrin, and leptin are being recognized as key mediators of angiogenesis and tumor growth (111). These pro-angiogenic factors play important roles both in maintenance and growth of the primary tumor as well as neo-vascularization during CCA metastasis (111). In normal tissues, following induction of angiogenesis by pro-angiogenic factors such as the VEGF factor family proteins (VEGF-A, VEGF-B, VEGF-C), remodeling of the newly formed vessel wall takes place where intercellular tight junctions and adherens junctions are created between vascular endothelial cells (BECs), that brings about permeability and elasticity in the vessel (110). After vessel remodeling is completed in normal tissues the ensuing blood flow and establishment of normoxia (normal/physiological O_2_ concentration) results in the inhibition of angiogenesis inhibition. In tumor cells however, hypoxia or a low oxygen environment in the region of the tumor induces expression of VEGF hormones. Cholangiocytes, accordingly, have been found to secrete high levels of both VEGF-A in the tumor stroma and VEGFR-2 during cholangiocyte hyperplasia (112). This suggests an autocrine mechanism by which cholangiocytes regulate their own growth. Similar to the studies in CCA lymphangiogenesis where high VEGFR-3 expression is enhanced under the influence of CAFs and tumor cells on the surface of LECs, it has been shown that a similar paracrine signaling mechanism exists in BECs where high levels of VEGFR-2 are expressed on its surface (112). Enhanced expression of VEGF-A and other members of the VEGF family such as VEGF-C cause BECs to secrete MMP-9 and MMP-7 which help in remodeling of the basement membrane and surrounding ECM and promotes tumor metastasis. Interestingly, it has been shown that TGF-β and VEGF are co-expressed in human CCA and that overexpression and functional interaction of TGF-β and VEGF could potentially contribute to the “angiogenic switch” and the malignant phenotype in human CCC (113). In addition to hypoxia stimulating production of VEGF, additional factors such as estrogen along with IGF1 (insulin like growth factor 1) and IGFR (IGF1 receptor) synergistically increases the expression of VEGFs such as VEGF-A, VEGF-C and their corresponding receptors in cultured CCA cells (114). In addition, metastasis-associated in colon cancer-1 (MACC1) protein upregulates VEGF-A thus favoring the growth of CCA (115). Overexpression of histidine decarboxylase (HDC) enzyme correlated with that of VEGF-A/C expression. HDC knockdown/inhibition significantly reduced tumor growth by reducing tumor cell proliferation and VEGF expression (109).

### microRNA Regulation of Lymphangiogenesis and Angiogenesis in CCA

Growing evidence from literature suggests that microRNAs (miRNA), endogenous small non-coding RNAs (19–24 nucleotides) regulate various aspects of cholangiopathies including CCA and has been extensively reviewed elsewhere (116, 117). However, studies evaluating their role in regulation of lymphangiogenesis associated with CCA is very limited. The miRNAs involved in CCA associated angiogenesis have been more extensively investigated and miR-92a, miR-126, miR-132, and miR-296 regulate several key pathways that enhance CCA associated angiogenesis (118). Overexpression of miR 16 and miR-424 has been shown to regulate the VEGF-A/FGF signaling cascades and reduce tumor cell proliferation and migration (119). miR-101, an miRNA highly expressed in liver was found to inhibit the growth of CCA by inhibiting VEGF expression (120). Understanding how miRNA regulate different molecular players involved at different levels of CCA progression also will help design better therapeutic interventions for arresting tumor progression. Further these miRNAs have the potential of being diagnostic biomarkers for CCA metastasis.

## Conclusion and Future Directions

**Figure 1:**
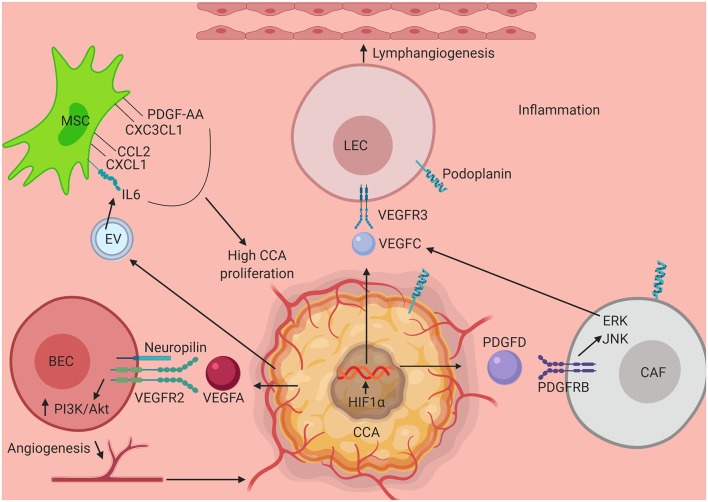
Schematic illustrating interaction of key elements in the tumor stroma in CCA progression. Several components of the CCA tumor microenvironment activate mechanisms that promote tumor growth, migration and activation of tumor associated angiogenesis and lymphangiogenesis. Interaction between VEC and CCA cell via VEGFR2-VEGFA assisted by neuropilin leads to tumor angiogenesis via upregulation of PI3K/Akt pathway. HIF1α activated by an inflamed tumor microenvironment stimulates CCA progression. VEGFC secreted by CCA as well by CAF (via PDGFD stimulation of tumor cells) aids in the process of lymphangiogenesis by stimulating LECs to divide via VEGFR3 engagement and upregulation of ERK/JNK pathway. Further contributing to the surrounding milieu, exosomal vesicles secreted by CCA triggers production of IL6, CCL2, CXCL1, CXC3CL1, and PDGF-AA which in turn when secreted in the tumor stroma induces CCA proliferation and growth pathways. CCA, Cholangiocarcinoma; MSC, Mesenchymal Stem Cell; LEC, Lymphatic Endothelial Cell; CAF, Cancer Associated Fibroblast; VEC, Vascular Endothelial Cell; PDGF-AA, Platelet Derived Growth Factor-AA; CXC3CL1, Chemokine Ligand 1 (Fractalkine); CCL2, C-C Motif Chemokine Ligand 2; CXCL1, C-X-C Motif Chemokine Ligand 1; IL6, Interleukin 6; VEGFR3, Vascular Endothelial Growth Factor Receptor 3; VEGFC, Vascular Endothelial Growth Factor C; VEGFA, Vascular Endothelial Growth Factor A; VEGFR2, Vascular Endothelial Growth Factor 2; PI3K, Phosphoinositide 3-kinase; Akt, Protein Kinase B; HIF1α, Hypoxia Inducible Factor 1 α; PDGFD, Platelet Derived Growth Factor D; PDGFRB, Platelet Derived Growth Factor Receptor B; ERK, Extracellular Receptor Kinase; JNK, c-Jun N-terminal Kinase.

The epidemiology of CCA varies across different regions owing to the differences in the number and intensity of the risk factors present in each place, the malignancy also varies in terms of the epidemiology of its types (iCCA, pCCA, dCCA), however based on the data above it can be postulated that inflammation of the tumor microenvironment and its associated players have a crucial role in shaping the response of the CCA cells to therapeutic strategies, their growth and progression. To this end, the early metastatic events of CCA is an area that can be pursued in the future to look for new therapeutic targets as well as to unravel the intricacies of the inflammatory tumor microenvironment-CCA crosstalk. While therapies targeting specific molecules and signaling pathways have shown promise, combinatorial therapies as a whole have come up to be effective in different cancer types. Hence, a better understanding of the different components of the tumor stroma that the CCA cells modulate and exploit in order to give rise to a pro-inflammatory and pro-tumorigenic environment can lead to a holistic understanding and approach toward treating CCA. Some of these key mechanisms that interact and promote the onset and progression and subsequent metastasis of CCA is shown in Figure 1. It is also evident that the aggressiveness of this cancer is directly related to its ability to metastasize and hence understanding key events that promote lymphatic metastasis in the early stages of the cancer will be critical for development of targeted therapies. Specific traits of CCA such as the high rate of lymphangiogenesis vs. the low rate of angiogenesis, deserve special research focus to unravel some of the underlying molecular pathways that mediate disease progression.

## Author Contributions

SR, SG, and SC planned and wrote the manuscript and approved the final submitted version. SR made the figure.

### Conflict of Interest

The authors declare that the research was conducted in the absence of any commercial or financial relationships that could be construed as a potential conflict of interest.
